# Emphysematous Cholecystitis and Necrotizing Pancreatitis: Unusual Combination of Critical Pathology in a Healthy Young Patient

**DOI:** 10.7759/cureus.9843

**Published:** 2020-08-18

**Authors:** Ahmad Abu Amr, Mohannad Al-Tarakji, Fakhar Shahid, Sania Razzaq, Syed Muhammad Ali

**Affiliations:** 1 General Surgery, Hamad Medical Corporation, Doha, QAT; 2 Acute Care Surgery, Hamad Medical Corporation, Doha, QAT; 3 Radiology, Hamad General Hospital, Doha, QAT; 4 Surgery, Weill Cornell Medical School, Doha, QAT; 5 Acute Care Surgery, Hamad General Hospital, Doha, QAT

**Keywords:** emphysematous cholecystitis, laparoscopic cholecystectomy, necrotizing pancreatitis

## Abstract

Emphysematous cholecystitis (EC) is a rare form of severe cholecystitis, more common among old, frail males, with comorbidities like diabetes mellitus and peripheral vascular disease. The coexistence of emphysematous cholecystitis with necrotizing pancreatitis is extremely uncommon and in the literature, though it has been reported, no case was of a young patient like the one we are describing. He was treated with laparoscopic cholecystectomy, and his pancreatitis managed conservatively.

## Introduction

Emphysematous cholecystitis (EC) is defined as inflammation of the gallbladder (GB), with the presence of air in the wall or lumen or adjacent to it without communication between the gallbladder and the hollow viscus, along with organ dysfunction [1]. Most often, the causative organisms are Escherichia coli and Clostridium species [2-3]. Concomitant necrotizing pancreatitis, which is pancreatitis and necrotic fluid collection around the pancreas or pancreatic parenchymal necrosis, with EC is rarely seen and usually thought to be a fatal combination. Necrotizing pancreatitis (NP) is usually detected with imaging like computed tomography (CT), according to the Atlanta classification [4]. The diagnosis depends mainly on clinical findings and imaging, and management is planned for whether a surgical intervention, which is an ideal or conservative approach with antibiotics and image-guided drainage, will be optimum.

## Case presentation

A 21-year-old gentleman previously healthy, non-smoker, was admitted through the emergency department with upper abdominal pain in the epigastric and right hypochondrium, which was of acute onset, progressive, sharp, radiating to the back, and associated with nausea and three episodes of vomiting. The pain started one day earlier, and he didn’t have chills or fever and there was no change in bowel or urinary function. He did not have any chronic illnesses and no history of familial disease. His heart rate was 90 beats/min, blood pressure 120/85 mmHg, and he had a normal body temperature of 36.8°C.

**Figure 1:**
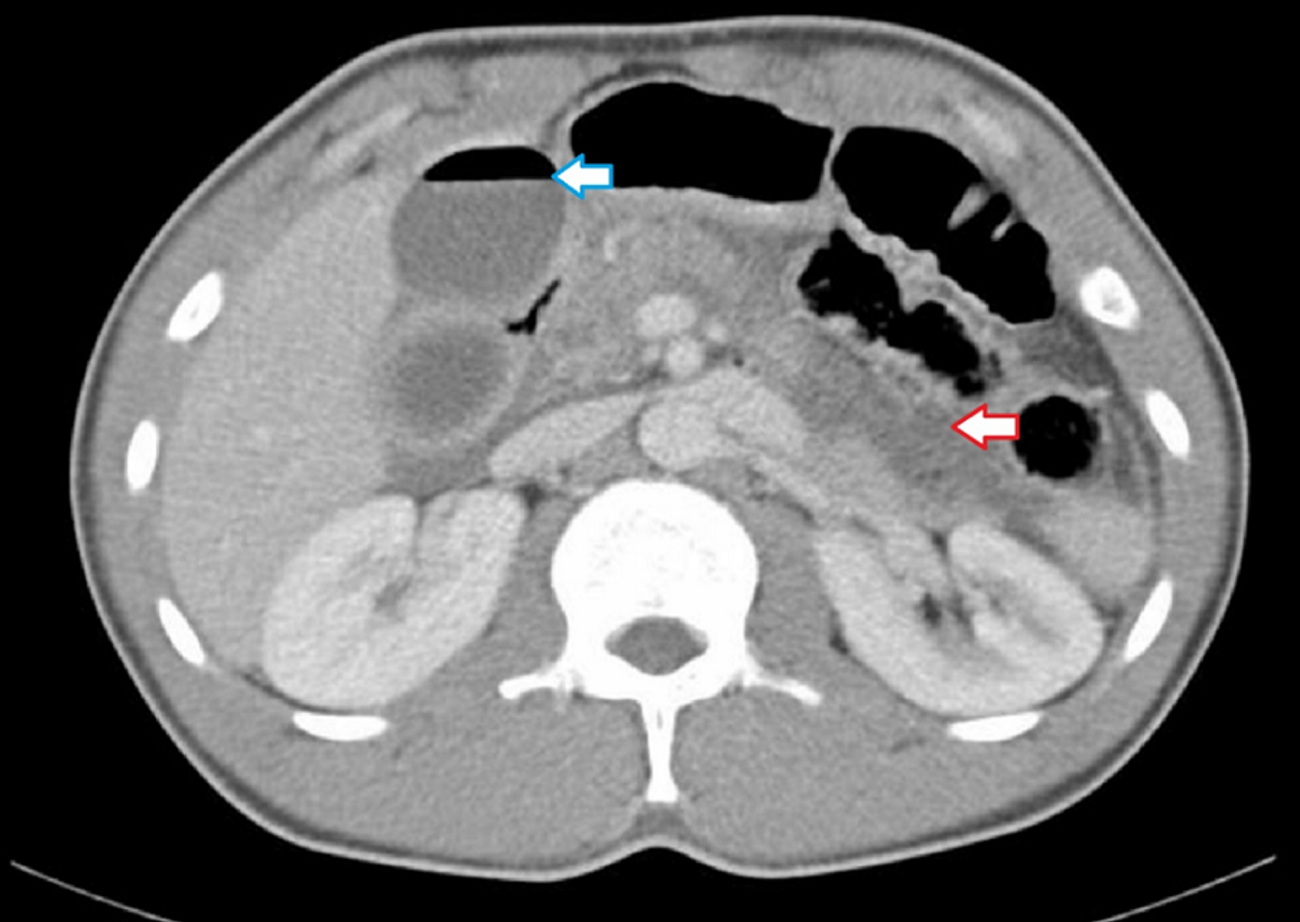
CT scan of the abdomen showing gas in the gallbladder (blue arrow) and necrosis involving the pancreas (red arrow) CT: computed tomography

On inspection, he had normal skin, a flat abdomen moving with respiration, although it was a little restricted in upper regions. The physical examination showed generalized guarding with rigidity especially in the epigastric and right upper quadrant regions along with marked tenderness. However, no mass was palpable in the right hypochondrium, possibly because of tenderness and guarding. Laboratory investigations showed a white cell count of 10.5/mm^3^ with left shift, hemoglobin 15.7 g/dL, lipase 4,383 U/L, and amylase 316 U/L. His liver enzymes and serum bilirubin were within the normal range whereas procalcitonin was 12.16 ng/mL (normal 0.10-0.49) and C-reactive protein (CRP) was 94 mg/dL (normal 0-10). Serum lactate was normal 0.5 mmol/L (normal 0.5-2.2). Ultrasound abdomen showed inflamed gallbladder with multiple small stones, 5-7 mm in size, with air (pneumobilia) and a bulky pancreas. The common bile duct (CBD) was 5 mm in diameter without intra-hepatic biliary dilatation. CT abdomen showed acute necrotizing pancreatitis involving the pancreatic head and body, the gallbladder appears to have an hour-glass configuration/septated with two separate cystic chambers. The gallbladder was moderately dilated with mild diffuse wall thickening, multiple 5-7 mm calculi in its lumen, and minimal pericholecystic collection along with air and air-fluid levels within the lumen (pneumobilia) (Figure 1).

Tiny specks of air were also noted within the gallbladder wall. Its wall adjoining the pancreatic head is significantly thinned out, suggestive of peripancreatic necrosis involving the gallbladder, acute emphysematous cholecystitis, and minimal ascites. The patient was nursed in the surgical intensive care unit (ICU) due to the risk of rapid deterioration with the diagnosis and CT findings. He was started on meropenem and blood culture showed no bacterial growth of any organism. After fluid administration and stabilization of vital signs, he underwent laparoscopic cholecystectomy with findings of an acutely inflamed gallbladder with fibrinous adhesions and pus collection in the Morison pouch and pelvis. Surgery was uneventful and the pus culture from the bile grew E. coli sensitive to cephalosporin, and it was changed accordingly. Histopathology of the gallbladder reported acute on chronic cholecystitis and necrosis of the wall without malignancy.

**Figure 2:**
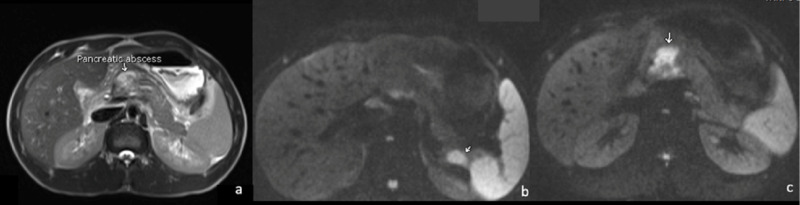
MRCP scan of the abdomen Multifocal pancreatic abscesses in multiple axial sequences as hyperintense in T2 (a), demonstrating diffusion restriction in DWI (b), and (c) located at the tail of the pancreas (annotated with an arrow) and another at the junction of the body and head of the pancreas MRCP: magnetic resonant cholangiopancreatography; DWI: diffusion-weighted imaging

He stayed two days in the surgical ICU and was shifted to the floor thereafter. He was continued on antibiotics for seven days and managed for pancreatitis. As he continued to have intermittent abdominal pain in the epigastric region, even after a week of cholecystectomy, magnetic resonant cholangiopancreatography (MRCP) was done to visualize the biliary system and the pancreas although his liver functions and bilirubin were normal. It showed an inflamed choledochal cyst of the distal part of the CBD and small stones, along with necrotizing pancreatitis with multiple abscesses not amenable to drainage (Figure 2).

**Figure 3:**
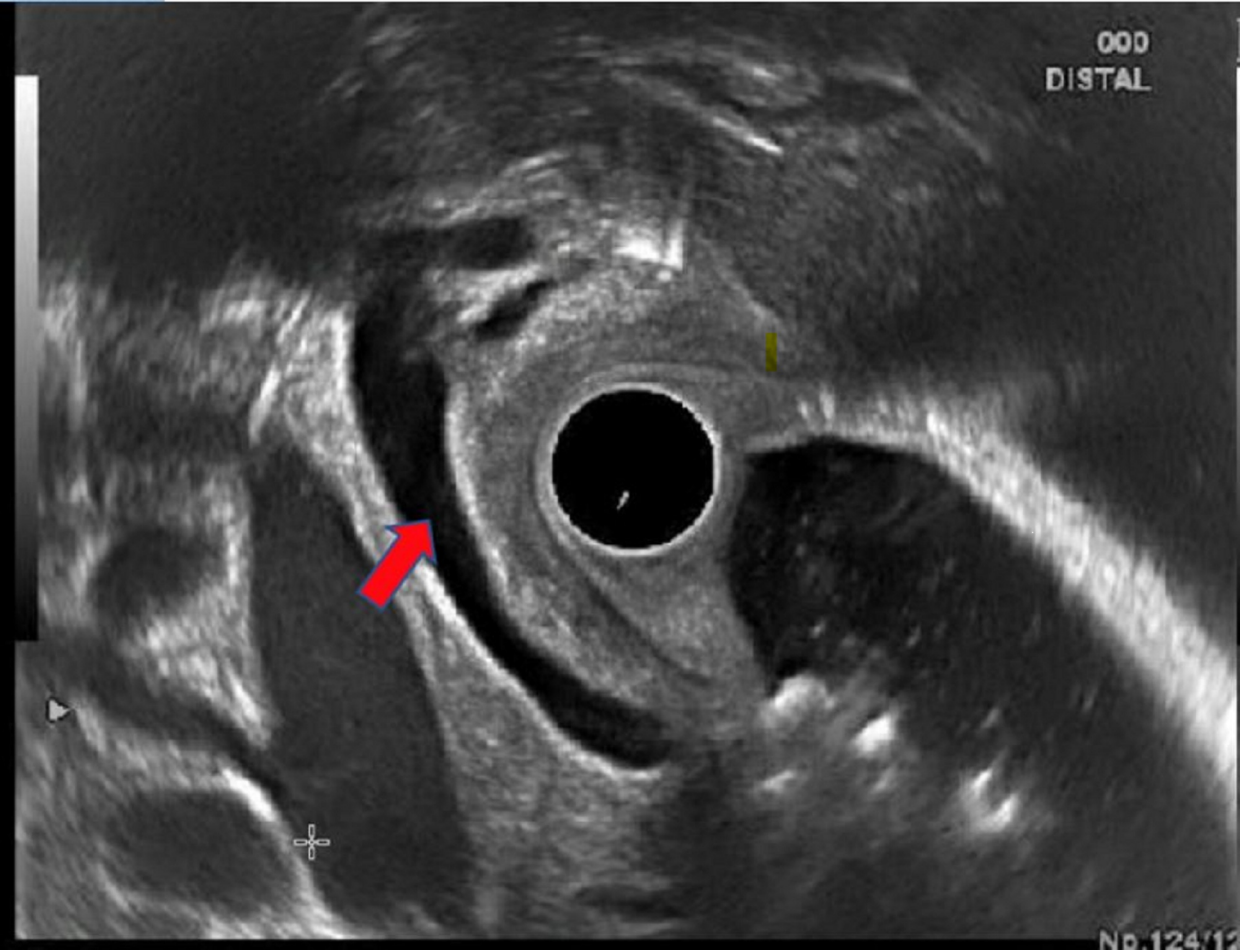
Endoscopic ultrasound image showing anechoic common bile duct (red arrow) measuring 5.8 mm with no evidence of calculi

Endoscopic retrograde cholangiopancreatography (ERCP) was planned to remove the stones in the lower end of the CBD having a choledochal cyst. However, it was not carried out, as endoscopic ultrasound (EUS) prior to ERCP did not show any choledochal cyst or common bile duct stones (Figure 3).

The patient improved significantly over the ensuing days and was discharged two weeks after admission in good general condition. He was followed up in the clinic at one and three months with no complaints or complications and normal liver function tests (LFTs).

## Discussion

EC is commonly seen in patients with comorbidities like diabetes and peripheral vascular disease. It represents 1%-3% of acute cholecystitis [1,5] and is more common in older men with a rate of 71% as compared to 27% of acute cholecystitis [2]. There are few proposed theories on the pathophysiology of EC formation: first, in critically ill patients, hypoperfusion to the gallbladder results in ischemia and the flourishing of gas-forming organisms [2,6]; second, there is obstruction of the cystic duct [7]. The mortality rate of EC is 1.4% in uncomplicated cases [8], and it reaches 25% if complicated [9].

The pathophysiology of the formation of gas in the GB is associated with organisms that are commonly isolated from the culture of the bile, including E. coli (40%), Bacteroides (30%), Clostridium species (25%), along with other organisms like P. vulgaris, A. aerogenes, Klebsiella, Streptococcus, Staphylococcus, and Enterococcus (40%) [3,10].

Necrotizing pancreatitis (NP) is a serious condition affecting the pancreas with a mortality rate ranging from 27-86% [11]. The combination of EC and NP has a grave prognosis and it is rare to see this combination. It would be expected in critically ill patients in the ICU setting with sepsis and systemic hypoperfusion without evidence of gallstones. However, pancreatitis in our case was due to a slipped gallstone to the CBD. The stone got impacted at the ampulla of Vater, as the patient had multiple small stones ranging from 5-7 mm, demonstrated by ultrasound examination. These small stones can get their way in the CBD and cause gallstone pancreatitis. Translocation of gas-forming bacteria like Clostridium perfringens and Escherichia coli to the gallbladder leads to air in the gallbladder and necrosis in the peripancreatic tissue [10].

The clinical presentation of these patients is similar to acute cholecystitis, with elevated liver function, jaundice, and fever. A high index of suspicion is kept in mind in the elderly with a compromised immune system or critically ill patients with organ hypoperfusion [5]. Ultrasound is highly sensitive and specific for identifying gallbladder disease and pathology [11], but in the case of EC combined with pancreatitis, ultrasound is inferior to CT, which is the most sensitive and specific imaging modality for the identification of gas in the wall, gallbladder lumen, and biliary ducts [8,12-13].

The treatment of choice for EC is laparoscopic cholecystectomy, with or without an intraoperative cholangiogram, which, however, could not be completed because of very difficult cystic duct cannulation during the on-table cholangiogram [10]. However, when the patient is in shock due to sepsis and anesthesia is relatively contraindicated owing to multiple comorbidities especially in the old, an image-guided cholecystostomy tube insertion for drainage is deemed appropriate along with antibiotics [14]. Cholecystectomy can be performed at a later stage until they are fit for surgical intervention. During our literature review, we found a similar reported case by Zuur et al. of an older man who succumbed to the disease [15].

## Conclusions

Emphysematous cholecystitis along with necrotizing pancreatitis is a rare combination of critical diseases affecting the elderly population with multiple comorbidities and carries a poor prognosis if not recognized early and treated promptly. Ultrasound and CT abdomen play an important role in the diagnosis and commencement of aggressive therapy. Laparoscopic cholecystectomy is the treatment of choice if the patient is fit to undergo surgical management, whereas cholecystostomy under radiological guidance drains the gallbladder efficiently in moribund and frail patients. The treatment of necrotizing pancreatitis is non-operative, and intervention is needed if there are complications like an abscess, extensive necrosis, or pseudocyst formation.

## References

[1] Wu CS, Yao WJ, Hsiao CH (1998). Effervescent gallbladder: sonographic findings in emphysematous cholecystitis. J Clin Ultrasound.

[2] Mentzer RM Jr, Golden GT, Chandler JG, Horsley JS 3rd (1975). A comparative appraisal of emphysematous cholecystitis. Am J Surg.

[3] Garcia-Sancho Tellez L, Rodriguez-Montes JA, Fernandez de Lis S, Garcia- Sancho Martin L (1999). Acute emphysematous cholecystitis. Report of twenty cases. Hepatogastroenterology.

[4] Banks PA, Bollen TL, Dervenis C (2013). Acute Pancreatitis Classification Working Group. Classification of acute pancreatitis—2012: revision of the Atlanta classification and definitions by international consensus. Gut.

[5] Choi HS, Lee YS, Park SB, Yoon Y (2010). Simultaneous emphysematous cholecystitis and emphysematous pancreatitis: a case report. Clin Imaging.

[6] May RE, Strong R (1971). Acute emphysematous cholecystitis. Br J Surg.

[7] Lorenz RW, Steffen HM (1990). Emphysematous cholecystitis: diagnostic problems and differential diagnosis of gallbladder gas collection. Hepatogastroenterology.

[8] Sunnapwar A, Raut AA, Nagar AM, Katre R (2011). Emphysematous cholecystitis: Imaging findings in nine patients. Indian J Radiol Imaging.

[9] Katagiri H, Yoshinaga Y, Kanda Y, Mizokami K (2014). Emphysematous cholecystitis successfully treated by laparoscopic surgery. J Surg Case Rep.

[10] Hazey JW, Brody FJ, Rosenblatt SM, Brodsky J, Malm J, Ponsky JL (2001). Laparoscopic management and clinical outcome of emphysematous cholecystitis. Surg Endosc.

[11] Negrão De Figueiredo G, Mueller-Peltzer K, Zengel P, Armbruster M, Rübenthaler J, Clevert DA (2019). Contrast-enhanced ultrasound (CEUS) and gallbladder diseases - a retrospective mono-center analysis of imaging findings with histopathological correlation. Clin Hemorheol Microcirc.

[12] Grayson DE, Abbott RM, Levy AD, Sherman PM (2002). Emphysematous infections of the abdomen and pelvis: a pictorial review. Radiographics.

[13] Bennet GL, Rusinek H, Lisi V, Israel GM, Krinsky GA, Slywotzky CM, Megibow A (2002). CT findings in acute gangrenous cholecystitis. AJR Am J Roentgenol.

[14] Fleming CA, Ismail M, Kavanagh RG (2020). Clinical and survival outcomes using percutaneous cholecystostomy tube alone or subsequent interval cholecystectomy to treat acute cholecystitis. J Gastrointest Surg.

[15] Zuur JK, Besselink MG, Mouwen DE (2014). Infected necrotising pancreatitis with associated emphysematous cholecystitis and pneumobilia. Netherlands J Crit Care.

